# London Medical Specialists
*"London Medical Specialists." By C. Bracebridge Allen, formerly of King's College. (London: Messrs. Ward, Lock,and Co.)


**Published:** 1890-07-26

**Authors:** 


### THE PRACTITIONER'S BOOKSHELF.

[Books for Review should be sent to The Editor, The Lodge, Porchester Square, W.]

### LONDON MEDICAL SPECIALISTS.*

"London Medical Specialists: A classified list of the names, addresses, &c., of all recognized authorities in the branches of medicine and surgery, with full information as to Special Hospitals." Such is the title page of this little book, and apart from its unprofessional character, we expected that at least the information it gave should be free from many omissions. We imagine the compiler has taken the staff lists of the special and other hospitals, and simply noted the names of the practitioners on them, and then has gone through the "Medical Directory" and placed on his list those names which have works on the special subjects attached to them. This may represent a considerable amount of diligence, but the book itself shows that the compiler has little working knowledge of the medical profession. For instance, under diseases of the bladder we see no mention of the name of Hill; diseases of the breast have surely had more attention paid to them than is conveyed in the bare mention of two names as authorities on the subject. Cancer claims the names of eight authorities ; but we think we could add a few to the list ; while diseases of the chest enumerates many, but omits quite as many. Has Roberts never done anything in this line of practice, or Godlee said nothing on empyæma? In contagious diseases we think the name of William Squire might appear, and Barker we always imagined had done some work in the field of aural surgery. Savage is generally considered an authority on mental disease, but we search in vain for the name in the list here given. We have said enough to show a number of omissions : the chief fault of commission is that the book was ever written, as the information required can always be obtained from any family doctor, or the pages of the recognized medical directories. We consider the list to be ill-advised, imperfect, and most assuredly invidious, and we doubt if anyone of recognized authority would countenance the continuance of its publication.

* "London Medical Specialists." By C. Bracebridge Allen, formerly of King's College. (London: Messrs. Ward, Lock, and Co.)

